# Translocation of gasdermin D induced mitochondrial injury and mitophagy mediated quality control in lipopolysaccharide related cardiomyocyte injury

**DOI:** 10.1002/ctm2.1002

**Published:** 2022-08-28

**Authors:** Ziqing Yu, Zilong Xiao, Lichun Guan, Pei Bao, Yong Yu, Yixiu Liang, Minghui Li, Zhenzhen Huang, Xueying Chen, Ruizhen Chen, Yangang Su, Junbo Ge

**Affiliations:** ^1^ Department of Cardiology Shanghai Institute of Cardiovascular Diseases Zhongshan Hospital Fudan University Shanghai P. R. China; ^2^ Department of Cardiovascular Diseases Key Laboratory of Viral Heart Diseases Ministry of Public Health Shanghai Institute of Cardiovascular Diseases Zhongshan Hospital Fudan University Shanghai P. R. China; ^3^ Graduate School Shanghai Medical College Fudan University Shanghai P. R. China; ^4^ Department of Cardiovascular Surgery Shanghai General Hospital, Shanghai Jiao Tong University, School of Medicine Shanghai P. R. China

**Keywords:** autophagic flux, gasdermin D, inflammation, lipopolysaccharide, mitochondria, mitochondrial membrane potential, mitophagy

## Abstract

**Backgrounds:**

Inflammation underlies the mechanism of different kinds of heart disease. Cytoplasmic membrane localized N‐terminal fragment of gasdermin‐D (GSDMD‐N) could induce inflammatory injury to cardiomyocyte. However, effects and dynamic changes of GSDMD during the process of lipopolysaccharide (LPS) related inflammatory stress induced cardiomyocyte injury are barely elucidated to date. In this study, LPS related cardiomyocyte injury was investigated based on potential interaction of GSDMD‐N induced mitochondrial injury and mitophagy mediated mitochondria quality control.

**Methods:**

HL‐1 cardiomyocytes were treated with LPS and Nigericin to induce inflammatory stress. The dual‐fluorescence‐labelled GSDMD expressed HL‐1 cardiomyocytes were constructed to study the translocation of GSDMD. The mitochondrial membrane potential (MMP) was measured by JC‐1 staining. Mitophagy and autophagic flux were recorded by transmission electron microscopy and fluorescent image.

**Results:**

GSDMD‐N showed a time‐dependent pattern of translocation from mitochondria to cytoplasmic membrane under LPS and Nigericin induced inflammatory stress in HL‐1 cardiomyocytes. GSDMD‐N preferred to localize to mitochondria to permeablize its membrane and dissipate the MMP. This effect couldn't be reversed by cyclosporine‐A (mPTP inhibitor), indicating GSDMD‐N pores as alternative mechanism underlying MMP regulation, in addition to mitochondrial permeability transition pore (mPTP). Moreover, the combination between GSDMD‐N and autophagy related Microtubule Associated Protein 1 Light Chain 3 Beta (LC3B) was verified by co‐immunoprecipitation. Besides, mitophagy alleviating GSDMD‐N induced mitochondrial injury was proved by pre‐treatment of autophagy antagonist or agonist in GSDMD‐knock out or GSDMD‐overexpression cells. A time‐dependent pattern of GSDMD translocation and mitochondrial GSDMD targeted mitophagy were verified.

**Conclusion:**

Herein, our study confirmed a crosstalk between GSDMD‐N induced mitochondrial injury and mitophagy mediated mitochondria quality control during LPS related inflammation induced cardiomyocyte injury, which potentially facilitating the development of therapeutic target to myocardial inflammatory disease. Our findings support pharmaceutical intervention on enhancing autophagy or inhibiting GSDMD as potential target for inflammatory heart disease treatment.

## Graphical abstract

- Dual‐fluorescence labelled GSDMD (BFP‐M_GSDMD‐EYFP) as a novel tool to manifest dynamic changes of GSDMD;
- A time dependent pattern of subcellular localization of GSDMD during LPS related inflammation;
- Pore‐forming function of GSDMD‐N as a complementary mechanism of MMP repression;
- Pharmaceutical intervention on enhancing autophagy or inhibiting GSDMD as potential target for inflammatory heart disease treatment.

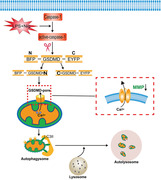

## 1 INTRODUCTION

Inflammation underlies in myocardial injury including ischemic injury, mechanical stress injury (pressure overload), metabolic disorder, infectious disease and myocarditis.^1^, ^2^ Lipopolysaccharide (LPS) related inflammatory injury could significantly influence the function and survival of cardiomyocytes. Besides, LPS induced cell inflammatory stress was reported to be mediated by gasdermin D (GSDMD) which directly leads to inflammatory pyroptosis.^3^, ^4^, ^5^ In inflammatory heart disease, GSDMD was regarded as one of the most important inflammatory effector molecule and could result in cardiomyocyte injury.^6^, ^7^ GSDMD, as a member of gasdermin protein family, could mediate pyroptosis in human and murine cells.^4^ GSDMD could be cleaved by inflammatory Caspase, for instance Caspase‐1, to generate an N‐terminal fragment product (GSDMD‐N) and C‐terminal fragment product (GSDMD‐C), during inflammation.^4^, ^5^, ^8^ In addition to Caspase‐1, Caspase‐3 was proved to cleave GSDME determined pyroptosis in certain GSDME‐expressing cancer cells.^9^ GSDMD‐C is thought to fold back on GSDMD‐N to inhibit its activation in full‐length GSDMD.^9^, ^10^ GSDMD‐N could localize to cell membrane and assemble into pore‐like structure to destroy the cellular integrity.^4^, ^10^, ^11^, ^12^ Mulvihill et al.,^13^ Ding et al.,^5^ and Liu et al.^4^ have captured the image of GSDMD pore, which define the GSDMD pore as oligomers with diameters of dozens of nanometre (nm). Highly expressed GSDMD is detected in mammalian myocardial tissue, indicating the importance of GSDMD in cardiac pathophysiology.^6^ GSDMD is reported to mediate HL‐1 cardiomyocyte injury by NLRP3 pathway.^6^ GSDMD‐knock out (GSDM‐KO) in cardiomyocyte could protect the mouse model from cardiac ischemia‐reperfusion injury.^6^, ^7^ However, the role of GSDMD in LPS related cardiomyocyte injury remains unclear.

The GSDMD‐N oligomerizes in cellular membranes to form pores, which is lethal to the cardiomyocyte. Considering some organelles such as mitochondria, Golgi body, endoplasmic reticulum and lysosome are coated by phospholipid bilayer,^14^ GSDMD‐N might permeablize the membrane of these organelles and influence their function maintenance. Recently, accumulated evidences disclosed that mitochondria localized GSDMD‐N was related to mitochondrial injury. Rogers et al. pointed out that GSDME‐N and GSDMD‐N could increase the permeability of the mitochondria to activate the mitochondrial apoptotic pathway.^15^ Huang et al. argued that mitochondria localized GSDMD‐N could mediate mitochondrial DNA leakage indicating increased mitochondrial membrane permeability.^16^ Another well‐acknowledged mechanism of mitochondrial injury based on increased mitochondrial membrane permeability is dissipated mitochondrial membrane potential (MMP) induced by mitochondrial permeability transition pore (mPTP) opening.^17^ Likewise, GSDMD pores could also make mitochondrial membrane permeable.^15^, ^16^ However, whether GSDMD related mitochondrial pores contribute to MMP like mPTP pores do has not been answered to date. Besides, it remains unclear about the translocation sequence of GSDMD‐N in response to LPS related inflammation in cardiomyocytes. The important work of de Vasconcelos et al. suggested the osmotic swelling of the whole cell and mitochondria occurred prior to sudden rupture of the plasma membrane.^18^ However, this study failed to further elucidate the specific spatiotemporal order of GSDMD‐N's subcellular localization. This might be partly owing to limited researching tool. Accordingly, a dual‐fluorescence labelled tool was built to investigate the translocation of GSDMD‐N in cardiomyocyte.

To our knowledge, GSDMD mediated mitochondria damage in inflammatory injury of cardiomyocyte has not been reported before. In this study, translocation of GSDMD‐N from cytosol to mitochondria during LPS related HL‐1 cardiomyocyte injury is investigated. Besides, we further discussed the presumed endogenous protective mechanism in the early phase of LPS related injury. Autophagy imposes great importance on cell quality control (QC).^19^ Elimination of damaged mitochondria by mitophagy is important to mitochondrial function maintenance.^20^, ^21^ Slightly activated mitophagy by inflammatory stress is reported in LPS‐induced septic cardiomyopathy.^22^ However, the role of mitophagy related mitochondria QC in GSDMD mediated mitochondrial injury has not been reported before. It is worthy of exploring whether microtubule associated proteins 1A/1B light chain 3B (LC3B) containing autophagosome could interact with the GSDMD‐N via a selective form of autophagy, namely mitophagy, to compensate mitochondrial damage in the early phase of LPS related injury. In the present study, we aim to disclose a crosstalk between mitophagy mediated mitochondrial QC and GSDMD‐N induced mitochondrial injury during inflammatory stress in cardiomyocyte. This study is designed to correlate with myocardial inflammatory disease seen in humans and could potentially facilitate the development of therapeutic target to myocardial inflammatory disease.

## 2 RESULTS

### 2.1 Blue fluorescence protein (BFP) linked GSDMD‐N as an indicator for the spatiotemporal specificity of mitochondria and cytoplasmic membrane localization during LPS related inflammatory stress

**FIGURE 1:**
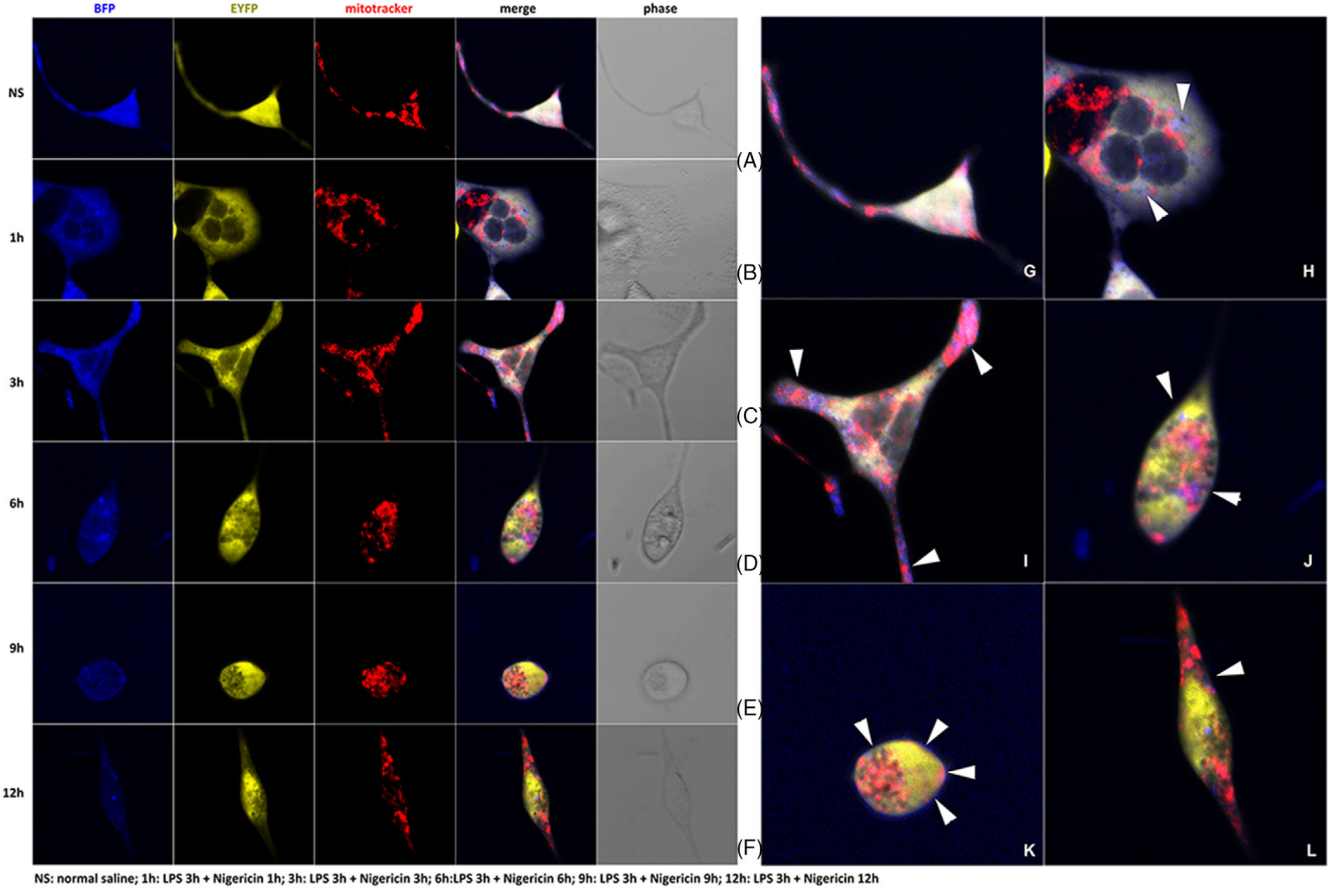
Time‐dependent pattern of blue fluorescence protein (BFP) linked GSDMD‐N translocation from mitochondria to cytoplasmic membrane during inflammatory stress. (A) Complete co‐localization of BFP and enhanced yellow fluorescence protein at baseline; (B) blue fluorescence separated from yellow fluorescence (1 h treatment of Nigericin after lipopolysaccharide [LPS]); (C) BFP‐GSDMD‐N begun to localize to mitochondria (3 h treatment of Nigericin after LPS); (D) significantly increased BFP‐GSDMD‐N fragment in mitochondria (6 h treatment of Nigericin after LPS); (E) cytoplasmic membrane localized BFP‐GSDMD‐N fragment (9 h treatment of Nigericin after LPS); (F) cytoplasmic membrane localized BFP‐GSDMD‐N fragment (12 h treatment of Nigericin after LPS); amplification of merged figure clearly indicating the translocation or marginalization of GSDMD‐N fragment at different phases; (G) intact GSDMD protein in cytosol at baseline; (H) cleaved GSDMD‐N fragment (blue) surrounding mitochondria (white arrow); (I) merged fluorescence (purple) suggesting co‐localization of GSDMD‐N and mitochondria (white arrow); (J) persistent mitochondria localized GSDMD‐N (purple) and marginalized GSDMD‐N (white arrow); (K) marginalized blue fluorescence suggesting cytoplasmic membrane localized GSDMD‐N fragment (white arrow); (L) intracellular blue fluorescence decay indicating a probably existed endogenous mechanism of GSDMD‐N elimination

Herein, the time‐dependent intracellular sub‐location of GSDMD‐N was investigated. Pre‐treatment of LPS for 3 h was applied to each intervention group to induce the priming condition of inflammatory stress. Five intervention protocols of subsequent Nigericin (1, 3, 6, 9 and 12 h, respectively) were allocated to cultured HL‐1 cells. At baseline, complete co‐localization of BFP and enhanced yellow fluorescence protein (EYFP) was observed without induction of GSDMD being cleaved (Figure 1A). However, the separation of blue and yellow fluorescence was observed after LPS treatment followed by Nigericin intervention in each cell group (Figure 1B–F), suggesting the formation of activated GSDMD fragments. After 1 h treatment of Nigericin, blue fluorescence separated from yellow fluorescence (Figure 1B). Interestingly, BFP‐GSDMD‐N localized to mitochondria (Figure 1C, purple colour) after 3 h of Nigericin treatment. At sixth hour, the intensity and area of merged purple fluorescence increased, indicating increased mitochondria localized BFP‐GSDMD‐N fragments within elongated intervention duration of Nigericin (Figure 1D). After persistent incubation with Nigericin for 9‐12 h, the cardiomyocyte was characterized with cell membrane marginalized blue fluorescence, indicating cytoplasmic membrane localized BFP‐GSDMD‐N fragment (Figure 1E,F). The amplified figures manifested the different spatiotemporal localization of BFP linked GSDMD‐N during LPS related inflammatory stress by the indication of white arrow heads (Figure 1G–L). In summary, translocation of GSDMD‐N fragment from cytosol to mitochondria and subsequently to cytoplasmic membrane was recorded in a time‐dependent manner during LPS plus Nigericin induced inflammatory stress.

Interestingly, the fluorescence of EYFP was concentrated rather than scattered in cytosol after the activation of GSDMD, indicating the accumulation of GSDMD‐C fragments (Figure 1C–F). We speculated that the aggregation of GSDMD‐C might facilitate the elimination of itself as a kind of non‐functional protein fragment. Furthermore, intracellular blue fluorescence decay was observed at time point longer than 6 h (Figure 1D,E), indicating the probable existence of endogenous mechanism of GSDMD‐N elimination. The investigation of presumed endogenous clearance of GSDMD‐N would be mentioned later.

### 2.2 Dynamic changes of mitophagy and its interaction with mitochondria localized GSDMD‐N

**FIGURE 2:**
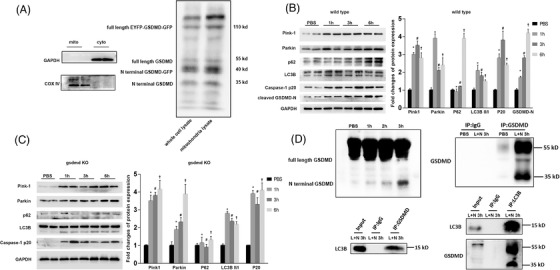
Dynamic changes of mitophagy and its interaction with mitochondria localized GSDMD‐N at protein level. (A) Isolated mitochondrial fraction and cytosolic fraction were verified by detection of cytosolic specific marker GAPDH and mitochondrial specific marker COX IV. Full length GSDMD, GSDMD‐N fragment, full‐length EYFP‐GSDMD‐GFP fusion protein, and GSDMD‐N‐GFP were detected in the separated fraction of whole cell lysate and mitochondrial fraction, respectively; (B) level changes (corrected by GAPDH level) of Pink‐1, Parkin, p62, LC3B II/I ratio, Caspase‐1 p20 and GSDMD‐N fragment after 1 h (*: *p* < .01, comparing with control), 3 h (#: *p* < .01, comparing with control), and 6 h (†: *p* < .01, comparing with control) of Nigericin (5 μM) intervention following LPS (1 mg/L) 3 h treatment in wild‐type HL‐1 cells (*p* < .01 by ANOVA, comparing among all groups); (C) level changes (corrected by GAPDH level) of Pink‐1, Parkin, p62, LC3B II/I ratio, Caspase‐1 p20 and GSDMD‐N fragment after 1 h (*: *p* < .01, comparing with control), 3 h (#: *p* < .01, comparing with control), and 6 h (†: *p* < .01, comparing with control) of Nigericin intervention following LPS 3 h treatment in GSDMD‐KO HL‐1 cells (*p* < .01 by ANOVA, comparing among all groups); (D) considering no GSDMD‐N fragments in saline treated HL‐1 cells, LPS 3 h plus Nigericin 1 h or Nigericin 2 h partly inducing GSDMD fragmentation, and LPS 3 h plus Nigericin 3 h completely inducing GSDMD fragmentation, LPS 3 h plus Nigericin 3 h was chosen as the intervention protocol for in‐input experiment for CO‐IP. In the step of input experiment, both full‐length GSDMD and its cleaved N‐terminal fragment were pulled down by themselves under induced inflammatory stress mentioned above. Then, on the one hand, LC3B was detected in GSDMD‐N combined fractions under inflammatory stress, indicating direct or indirect combination between LC3B and GSDMD. Finally, on the other hand, both GSDMD and its N‐terminal fragment was detected in LC3B combined fractions, which confirm the combination of LC3B and GSDMD

Mitochondrial fraction isolation was validated by detection of COX IV and no detection of GAPDH. BFP linked GSDMD‐N production induced by LPS related inflammation was verified in whole cell lysate. In addition, the separated fraction of mitochondria was isolated to detect full length and N‐terminal fragment of GSDMD (Figure 2A). The cleaved segment of BFP ligated GSDMD‐N was detected at about 60 Kd in mitochondria lysate after LPS 3 h plus Nigericin 3 h treatment (Figure 2A), which, either validating the mitochondrial localized GSDMD after inflammatory stimulation or verifying the efficacy of the dual‐fluorescence, labelled fusion protein as an indicator of GSDMD activation.

As mentioned above, blue fluorescence attenuation suggested the elimination of GSDMD‐N. We further investigated whether autophagy could function as basis of GSDMD‐N clearance. Based on our findings, we assumed that mitophagy might help to compensate the GSDMD‐N induced mitochondria injury. After 1, 3 and 6 h of Nigericin intervention following 3 h LPS treatment, levels of LC3B II/I ratio, Parkin and Pink1 all increased (Figure 2B). However, the expression of P62 was significantly elevated at 6 h suggesting restrained autophagic flux (Figure 2B). In contrast, P62 was decreased at 6 h, however, LC3B II/I ratio, Parkin and Pink1 still stayed in high level at three time points in GSDMD‐KO cells (Figure 2C), respectively. This hinted that depressed autophagic flux was partially restored by GSDMD ablagation. In both wild type (WT) cells and GSDMD‐KO cells, the active segment of Caspase‐1, Caspase‐1 p20, was elevated at each time point, which was in consistency with changes of GSDMD‐N level (Figure 2B,C). To testify whether the LC3‐autophagosome could target and envelop the mitochondria localized GSDMD N‐terminal fragment, co‐immunoprecipitation (CO‐IP) experiment was done to validate the interaction between LC3B and GSDMD. In input experiment, saline treated HL‐1 cells showed no GSDMD fragments, however, LPS 3 h plus Nigericin 3 h could induce GSDMD fragmentation (Figure 2D). In CO‐IP experiment, both full‐length GSDMD and its cleaved N‐terminal fragment were verified by Western blots (WB), indicating they could be pulled down by themselves (Figure 2D). LC3B was then detected in GSDMD‐N combined fractions (Figure 2D). Likewise, after pulling down LC3B combined fractions, both GSDMD and its N‐terminal fragment were detected (Figure 2D). Mutual authentication by CO‐IP method verified the interaction between LC3B and GSDMD‐N. We concluded that GSDMD‐N in mitochondria could recruit LC3B, and the latter one could facilitate to eliminate injured mitochondria resulted from effects of GSDMD. Interestingly, we found that either full‐length GSDMD or N‐terminal fragment could combine with LC3B. Based on previous results in this study, GSDMD and LC3B could co‐localize with mitochondria, respectively.

### 2.3 GSDMD influencing autophagic flux changes during LPS related inflammation

**FIGURE 3:**
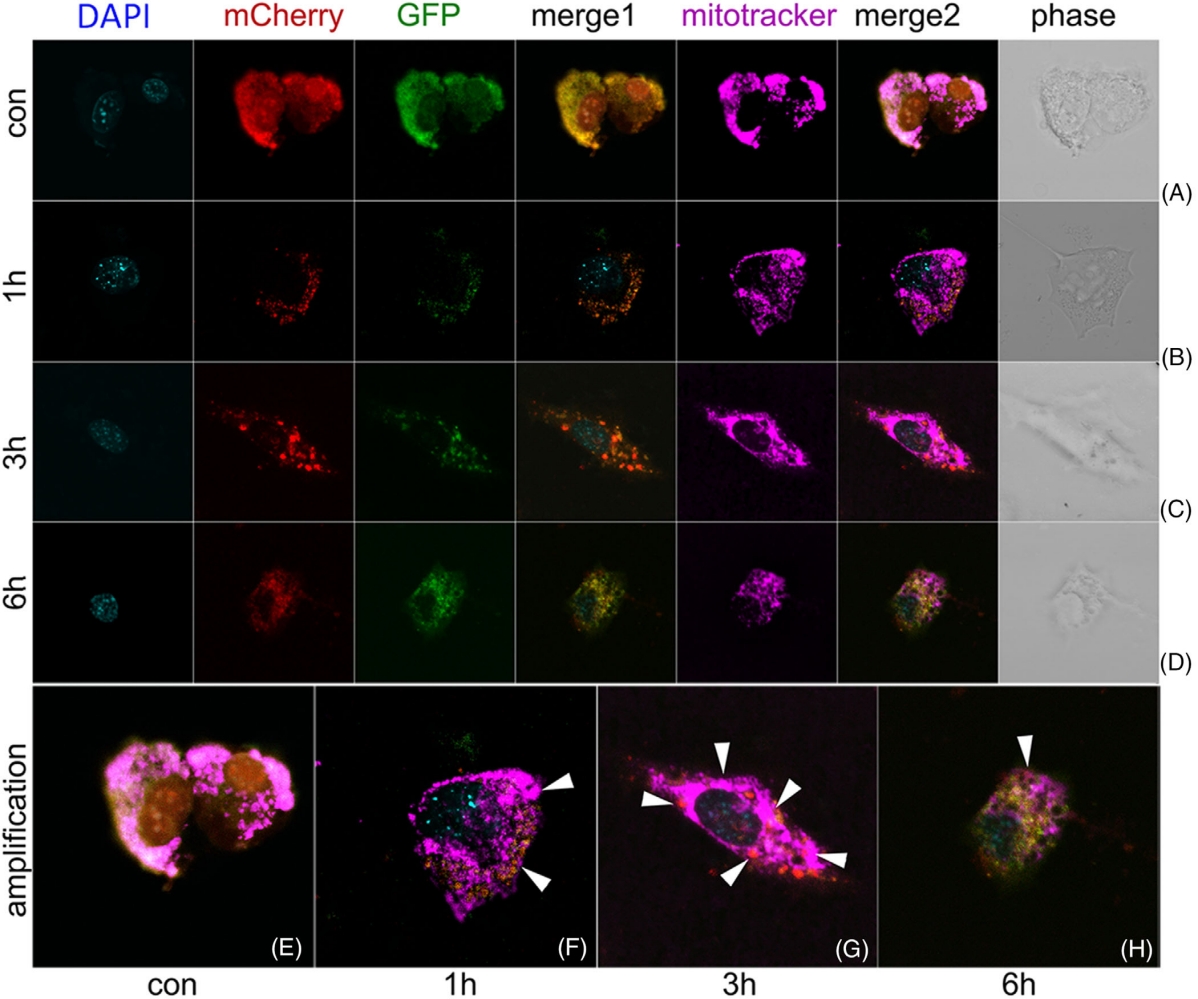
Dynamic changes of mitophagic flux under LPS related inflammation in wild‐type HL‐1 cells. (A) The fluorescence pattern of mCherry‐GFP‐LC3B at baseline with co‐staining of nuclei indicator DAPI, mitochondria indicator mitotracker; (B) significantly upregulated autophagic flux (increased punctate red fluorescence) after 1 h of Nigericin (5 μM) intervention following LPS (1 mg/L) 3 h treatment; (C) significantly upregulated autophagic flux (increased punctate red fluorescence) after 3 h of Nigericin (5 μM) intervention following LPS (1 mg/L) 3 h treatment; (D) decay of punctate red fluorescence revealing inhibited autophagic flux after 6 h of Nigericin (5 μM) intervention following LPS (1 mg/L) 3 h treatment; (E–H) amplification of merged figure clearly indicating the change of mitochondria autophagic flux at different phase; (E) mCherry‐GFP‐LC3B fluorescence pattern at baseline; (F) increased punctate red fluorescence indicating upregulated autophagic flux (white arrow) after 1 h of Nigericin (5 μM) intervention following LPS (1 mg/L) 3 h treatment; (G) increased punctate red fluorescence indicating upregulated autophagic flux (white arrow) after 3 h of Nigericin (5 μM) intervention following LPS (1 mg/L) 3 h treatment; (H) decay of punctate red fluorescence revealing inhibited autophagic flux (white arrow) after 6 h of Nigericin (5 μM) intervention following LPS (1 mg/L) 3 h treatment

**FIGURE 4:**
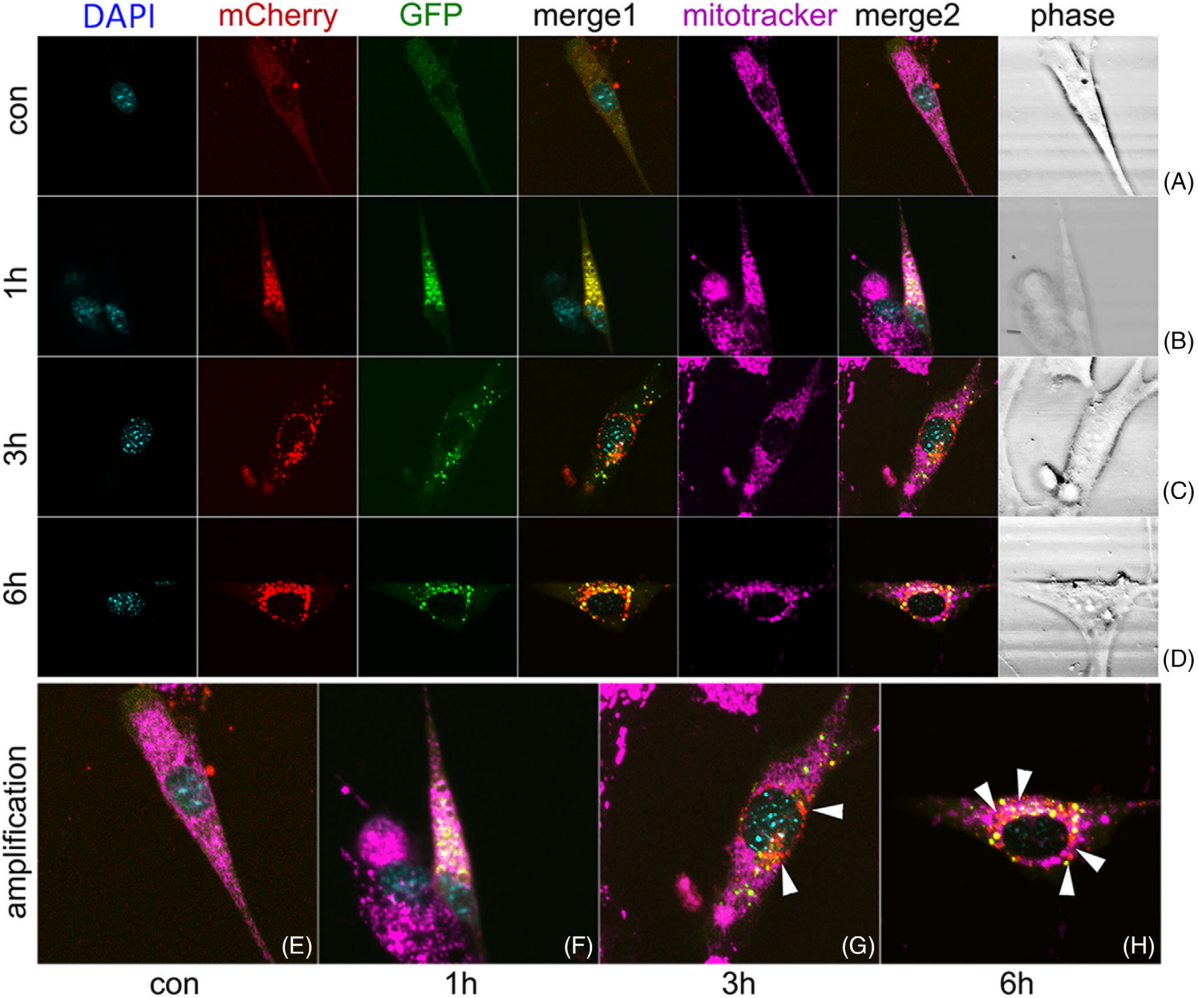
Dynamic changes of mitophagic flux under LPS related inflammation in GSDMD‐KO HL‐1 cells. (A) The fluorescence pattern of mCherry‐GFP‐LC3B at baseline with co‐staining of nuclei indicator DAPI, mitochondria indicator mitotracker; (B) significantly upregulated autophagic flux (increased punctate red fluorescence) after 1 h of Nigericin (5 μM) intervention following LPS (1 mg/L) 3 h treatment; (C) significantly upregulated autophagic flux (increased punctate red fluorescence) after 3 h of Nigericin (5 μM) intervention following LPS (1 mg/L) 3 h treatment; (D) persistently increased punctuate red fluorescence after 6 h of Nigericin (5 μM) intervention following LPS (1 mg/L) 3 h treatment indicating restored autophagic flux; (E–H) amplification of merged figure clearly indicating the change of mitochondria autophagic flux at different phase; (E) mCherry‐GFP‐LC3B fluorescence pattern at baseline; (F) increased punctate red fluorescence indicating upregulated autophagic flux after 1 h of Nigericin (5 μM) intervention following LPS (1 mg/L) 3 h treatment; (G) increased punctate red fluorescence indicating upregulated autophagic flux (white arrow) after 3 h of Nigericin (5 μM) intervention following LPS (1 mg/L) 3 h treatment; (H) persistently increased punctuate red fluorescence (white arrow) after 6 h of Nigericin (5 μM) intervention following LPS (1 mg/L) 3 h treatment indicating restored autophagic flux

Next, we testified whether GSDMD‐N could mediate LC3B anchoring to mitochondria and the effect of GSDMD‐N on autophagic flux. HL‐1 cells with WT genotype or GSDMD‐KO genotype were transfected with Ad‐mCherry‐GFP‐LC3B, a double fluorescence labelled LC3B sequence loaded by adenovirus to further disclose changes of autophagic flux before and after GSDMD ablation. Attenuation of green fluorescence and enhancement of red fluorescence manifested unobstructed autophagic flux. LC3B localized to mitochondria after LPS plus Nigericin treatment (Figure 3). In PBS treated cells, green fluorescence and red fluorescence were shown to distribute the target cell uniformly (Figure 3A). In accordance with changes of autophagy related proteins, autophagic flux was significantly upregulated (increased punctate red fluorescence) after 1 and 3 h of Nigericin intervention following LPS treatment (Figure 3B,C). After 6 h of Nigericin, punctate red fluorescence was decayed (Figure 3D), revealing inhibited autophagic flux. Changes of autophagic flux were similarly between WT HL‐1 cells and GSDMD‐KO HL‐1 cells at 1 and 3 h (Figure 4B,C). However, punctuate red fluorescence was increased in GSDMD‐KO HL‐1 cells comparing with WT HL‐1 cells at 6 h (Figure 4D), indicating partly restored autophagic flux. The amplified figures manifested the co‐localization between LC3B and mitochondria by the indication of white arrow heads (Figures 3E–H and 4E–H).

### 2.4 GSDMD related changes of mitochondria ultrastructure

**FIGURE 5:**
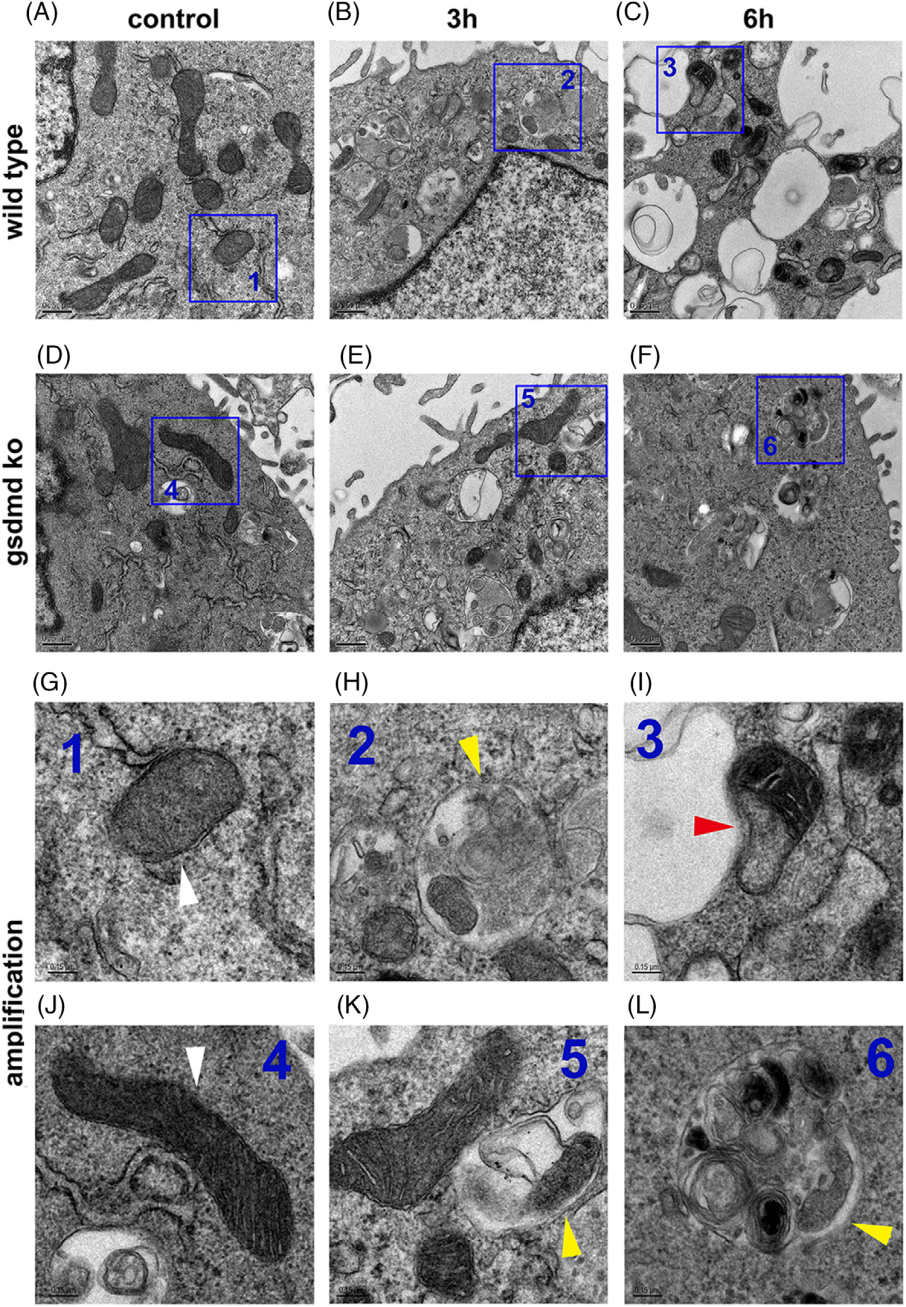
GSDMD correlating with mitochondria ultrastructural changes under LPS related inflammation. (A) Normal mitochondria morphology in normal saline treated wild‐type HL‐1 cells; (B) mild mitochondrial injury in accompany with significantly enhanced mitophagosome formation after the treatment of LPS (1 mg/L) 3 h plus Nigericin (5 μM) 3 h; (C) moderate to severe mitochondrial injury and significantly decreased mitophagosome and autophagolysosome; (D) normal mitochondria morphology in normal saline treated GSDMD‐KO HL‐1 cells; (E) improved mitochondrial morphology and increased autolysosome; (F) moderate mitochondrial injury and increased mitophagosome and autophagolysosome; (G–L) amplification of the region of interest in each figure; (G) normal mitochondria morphology (white arrow); (H) autolysosome with mitochondria debris inside it (yellow arrow); (I) injured mitochondria (red arrow); (J) normal mitochondria morphology (white arrow); (K) autolysosome with mitochondria debris inside it (yellow arrow); (L) autolysosome with mitochondria debris inside it (yellow arrow)

At baseline, mitochondria in control cells or GSDMD‐KO cells presented similar morphology (Figure 5A,D,G,J). In control cells, mild mitochondrial injury with significantly enhanced mitophagosome formation was observed after the treatment of 3 h LPS plus 3 h Nigericin (Figure 5B). Besides, increased autophagolysosome was simultaneously shown in Figure 5H. Moreover, after the treatment of LPS 3 h plus Nigericin 6 h, the degree of mitochondrial injury was ranging from moderate to severe (Figure 5C). Meanwhile, the autophagolysosome could hardly be observed in the same phase, in spite of the significant mitochondria damage (Figure 5I). Regardless of the accumulation of severely damaged mitochondria and mitophagosome, the autophagolysosome was scarcely found at the same time (Figure 5C,I), indicating a suppressed autophagic flux. On the contrary, GSDMD knocking‐out could improve mitochondrial morphology and partly restore the autophagic flux (Figure 5E,F,K,L). These transmission electron microscopy (TEM) findings along with above‐mentioned results collectively showed that GSDMD correlated with enhanced mitophagy in the early phase of LPS related inflammatory stimulation. However, GSDMD induced mitochondria injury resulted in prohibited autophagic flux with prolonged inflammatory stimulation, revealing a time‐dependent and double‐edged‐sword effect of GSDMD in mitochondrial QC and injury.

### 2.5 GSDMD‐N mediated mitochondrial membrane permeability underlying mitochondrial membrane potential repression

**FIGURE 6:**
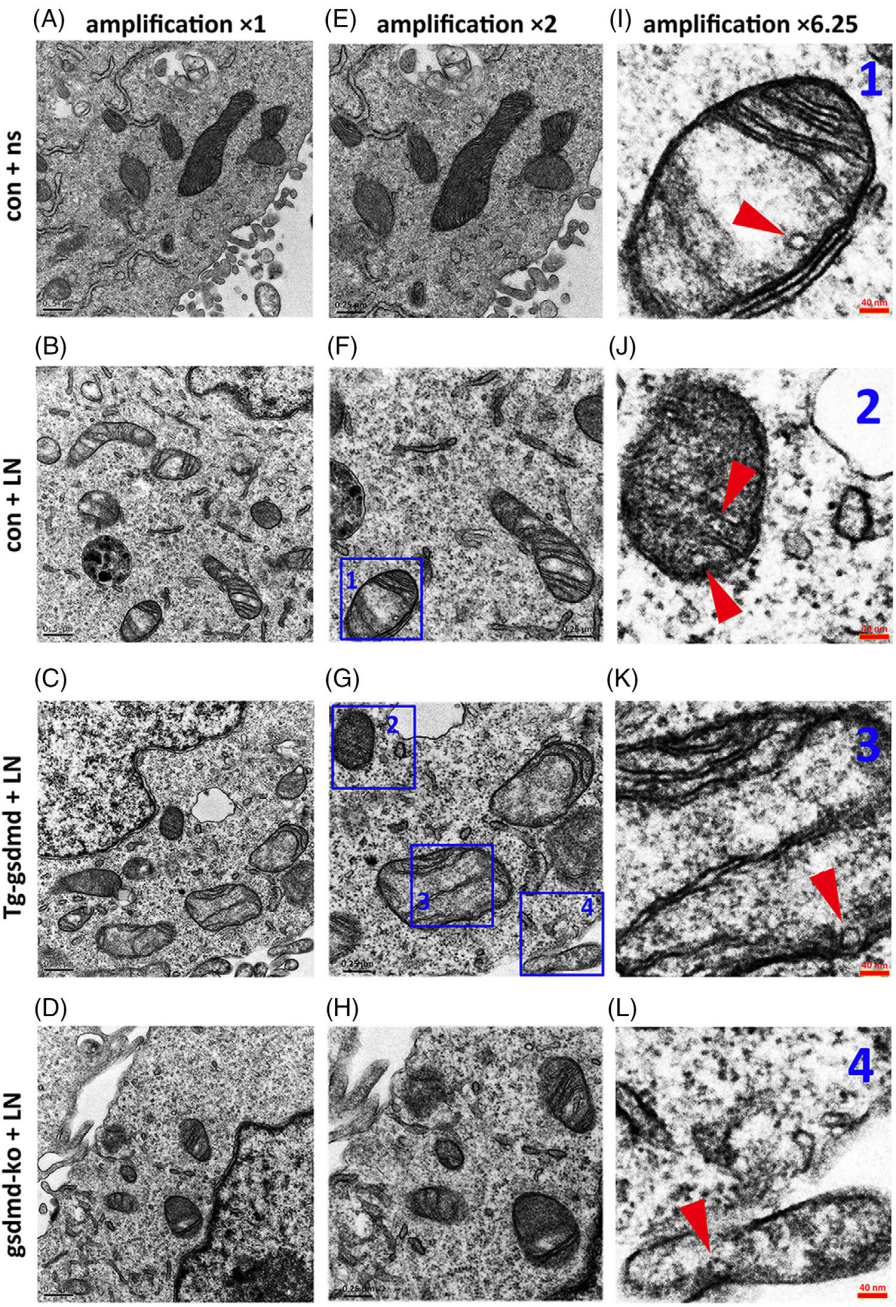
Time‐dependent changes of GSDMD related pore‐like structure in mitochondria. (A) Normal mitochondria morphology and intact mitochondria membrane in normal saline treated wild‐type HL‐1 cells; (B) well circumscribed pore‐like structure with diameter of 15–20 nm in mitochondria after the treatment of LPS (1 mg/L) 3 h plus Nigericin (5 μM) 3 h in wild‐type HL‐1 cells; (C) increased number of pore‐like structure with diameter of 15–20 nm in mitochondria after the treatment of LPS 3 h plus Nigericin 3 h in Tg‐GSDMD HL‐1 cells; (D) rare pore‐like structure formation in GSDMD‐KO HL‐1 cells with the existence of inflammatory stimulation; (E) normal mitochondria morphology and intact mitochondria membrane in normal saline treated GSDMD‐KO HL‐1 cells; (F) moderate mitochondrial injury and increased mitophagosome and autophagolysosome; (E–L) different magnifications of the region of interest in each figure to show the pore‐like structure in mitochondria (red arrowhead). GSDMD‐Tg, GSDMD transgenic; GSDMD‐KO, GSDMD knocking out

**FIGURE 7:**
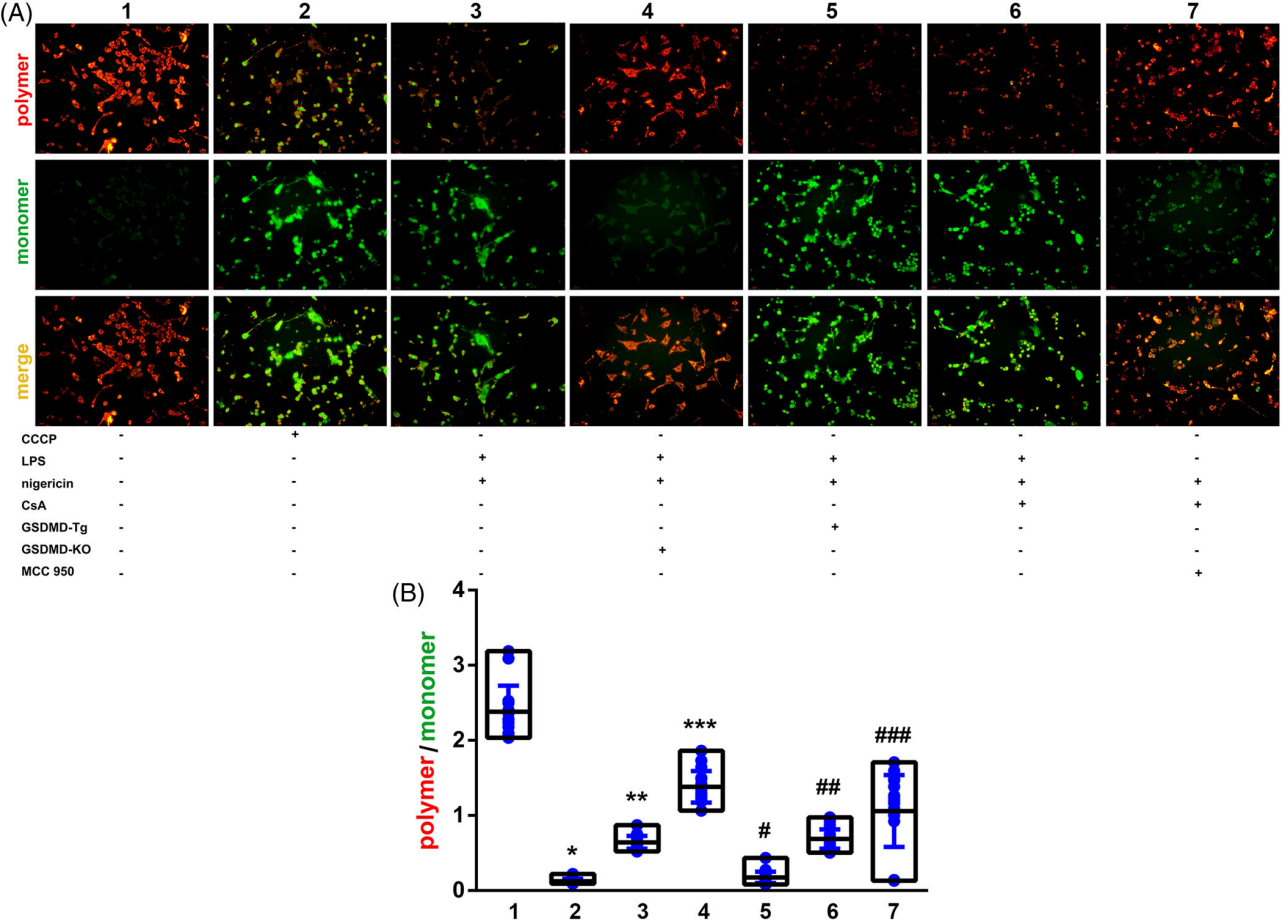
GSDMD related pore‐like structure mediating mitochondrial membrane potential dissipation during LPS related inflammation. (A) JC‐1 staining after different protocol of intervention (different combination of CCCP, LPS, Nigericin, CsA, GSDMD‐Tg, GSDMD‐KO, and MCC 950) to indicate changes of polymer and monomer fluorescence (polymer: red; monomer: green); (B) detection of the fluorescence intensity of JC‐1 polymer and JC‐1 monomer and calculating the polymer/monomer ratio by fluorescence microplate reader (*p* < .01 by ANOVA). CCCP group as positive control showed significant decreased MMP (*: *p* < .01, 2 vs. 1). The treatment of LPS (1 mg/L) 3 h plus Nigericin (5 μM) 3 h in wild‐type HL‐1 cells significantly induced MMP dissipation compared to control group (**: *p* < .01, 3 vs. 1). In GSDMD‐KO cells, MMP could be protected from severe destruction (***: *p* < .01, 4 vs. 3). On the contrary, in GSDMD‐Tg cells, MMP was further decreased (#: *p* < .01, 5 vs. 3). Pre‐treatment of CsA (50 nM), a specific inhibitor of mPTP pore, showed little protective effect on MMP maintenance under LPS related inflammatory stimulation (##: *p* > .05, 6 vs. 3), indicating a mechanism of mPTP independent and GSDMD related MMP regulation. Pre‐treatment of MCC950 (10 nM), a specific inhibitor of NLRP3 which lying in the upstream of GSDMD regulating pathway, showed significant protective effect on MMP maintenance under LPS related inflammatory stimulation (###: *p* < .01, 7 vs. 3), indicating indirectly targeting GSDMD could defend the harmful influence of LPS related inflammation to MMP

The morphological changes of mitochondria showed a time‐dependent characteristic. Mitochondrial morphology was normal and mitochondria membrane was intact in control group (Figure 6A,E). Compared to control group, well circumscribed pores with diameter of 15‐20 nm were observed in the surface of mitochondria (Figure 6B,F) in HL‐1 cells under inflammatory stress. Moreover, the pore structure was more frequently found in GSDMD overexpression cells (Figure 6C,G). On the contrary, mitochondrial membrane pore formation was hardly seen in GSDMD‐KO HL‐1 cells with or without the existence of LPS stimulation (Figure 6D,H), implying the suppressed formation of GSDMD pore in mitochondria. Single mitochondria image was amplified to show mitochondrial membrane pores (Figure 6I–L), and the diameter of theses pores were in accordance with the characteristics of GSDMD pores reported by Ding et al.^5^ and Liu et al.^4^ We hypothesized that mitochondrial GSDMD pores should be related to MMP fluctuating. Herein, the inflammatory stimulation protocol of LPS 3 h plus Nigericin 3 h was applied to investigate the function of GSDMD‐N in mitochondrial permeability and MMP maintenance. As is known to us, the mPTP opening is a well‐acknowledged mechanism underlying the dissipation of MMP,^17^ and the impaired MMP could lead to mitochondrial dysfunction, even severe damage. In this part, the mitochondria were proved to be permeabilized by GSDMD‐N. Carbonyl cyanide‐m‐chlorophenylhydrazone (CCCP) was used as positive control of decreased MMP. MMP was decreased in WT HL‐1 cell with LPS 3 h plus Nigericin 3 h treatment. In GSDMD‐KO HL‐1 cell, MMP could be maintained under inflammatory stimulation. In GSDMD‐Tg HL‐1 cell, MMP could be further impaired under inflammatory stimulation. To testify whether GSDND‐N related mitochondrial membrane pore could mediate the dissipation of MMP, WT HL‐1 cell were pre‐treated with mPTP inhibitor cyclosporine (CsA) for 6 h, and then LPS 3 h plus Nigericin 3 h could still induce significant MMP depression. Ultimately, GSDMD‐Tg HL‐1 cells were pre‐treated with MCC950, specific inhibitor of NLRP3 which is the upstream regulator of GSDMD activation, for 6 h. Afterwards, LPS 3 h plus Nigericin 3 h was treated, and the further impaired MMP in GSDMD‐Tg HL‐1 cells could be salvaged by MCC950 pre‐treatment (Figure 7A). In addition, the fluorescence intensity (polymer and monomer) of cultured cells incubated with JC‐1 stain was detected by fluorescence microplate reader. In accordance with the fluoroscopy (Figure 7A), the same results were attained in aforementioned cell groups (Figure 7B). LPS 3 h plus Nigericin 3 h could decrease the intensity of red fluorescence and increase the intensity of green fluorescence. Additionally, LPS 3 h plus Nigericin 3 h could further downregulate the MMP to a lower level in GSDMD‐Tg cells. Thoroughly knocking out GSDMD in vitro could radically protect mitochondria from dissipated MMP, indicating GSDMD‐N pore as an mPTP independent way of MMP regulation. After all, pre‐treatment of CsA could slightly mitigate the anomaly of MMP induced by LPS 3 h plus Nigericin 3 h. Besides, pre‐treatment of MCC950, a specific inhibitor of NLRP3 which is the upstream signal molecule of GSDMD could significantly protect mitochondria from MMP impairment in GSDMD‐Tg cell. Both results of MMP measurement by JC‐1 staining and mitochondrial morphology study by TEM suggested that mitochondrial QC should correlate with mitophagy and autophagic flux. Furthermore, the relationship between GSDMD and mitophagy during LPS related inflammation was validated.

### 2.6 Enhancement of mitophagy decreasing cytotoxicity and mitochondrial membrane potential during LPS related inflammation

**FIGURE 8:**
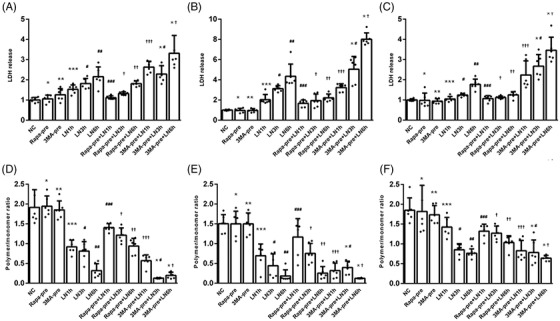
Enhancement of mitophagy decreasing cytotoxicity and stabilizing mitochondrial membrane potential during LPS related inflammation. LDH release detection after different protocol of intervention indicated changes of cytotoxicity in wild‐type HL‐1 cells with different intervention group (*p* < .01 by ANOVA). Pre‐treatment of Rapa (1 μmol/L) for 6 h did not influence cytotoxicity (*: *p* > .05, Rapa‐pre vs. NC). Likewise, pre‐treatment of 3‐MA (50 μM) for 6 h did not influence cytotoxicity (**: *p* > .05, 3‐MA‐pre vs. NC). Comparing with NC cells, Nigericin (5 μM) for 1, 3, and 6 h after LPS (1 mg/L) resulted in time‐dependent cytotoxicity increase (***: *p* < .01; #: *p* < .01; ##: *p* < .01). Pre‐treatment of Rapa could significantly alleviate cytotoxicity induced by the LPS related inflammation (###: *p* < .01, Rapa‐pre + LN 1 h vs. LN 1 h; †: *p* < .01, Rapa‐pre + LN 3 h vs. LN 3 h; ††: *p* < .01, Rapa‐pre + LN 6 h vs. LN 6 h). Oppositely, pre‐treatment of 3‐MA could significantly aggravate cytotoxicity induced by the LPS related inflammation (†††: *p* < .01, 3‐MA‐pre + LN 1 h vs. LN 1 h; *#: *p* < .01, 3‐MA‐pre + LN 3 h vs. LN 3 h; *†: *p* < .01, 3‐MA‐pre + LN 6 h vs. LN 6 h); (B) LDH release detection after different protocol of intervention indicated changes of cytotoxicity in Tg‐GSDMD HL‐1 cells with different intervention group (*p* < .01 by ANOVA). Pre‐treatment of Rapa or 3‐MA did not influence cytotoxicity (*: *p* > .05, Rapa‐pre vs. NC; **: *p* > .05, 3‐MA‐pre vs. NC). Comparing with NC cells, Nigericin for 1, 3, and 6 h after LPS gradually induced escalation of cytotoxicity (***: *p* < .01; #: *p* < .01; ##: *p* < .01). Pre‐treatment of Rapa could partly alleviate cytotoxicity induced by the LPS related inflammation (###: *p* < .01, Rapa‐pre + LN 1 h vs. LN 1 h; †: *p* < .01, Rapa‐pre + LN 3 h vs. LN 3 h; ††: *p* < .01, Rapa‐pre + LN 6 h vs. LN 6 h). Oppositely, pre‐treatment of 3‐MA could further aggravate cytotoxicity induced by the LPS related inflammation (†††: *p* < .01, 3‐MA‐pre + LN 1 h vs. LN 1 h; *#: *p* < .01, 3‐MA‐pre + LN 3 h vs. LN 3 h; *†: *p* < .01, 3‐MA‐pre + LN 6 h vs. LN 6 h); (C) in GSDMD‐KO cells, pre‐treatment of Rapa or 3‐MA did not influence cytotoxicity (*: *p* > .05, Rapa‐pre vs. NC; **: *p* > .05, 3‐MA‐pre vs. NC). Comparing with NC cells, the trend of LN treatment induced cytotoxicity for 1, 3, and 6 h was observed (***: *p* < .01; #: *p* < .01; ##: *p* < .01). Pre‐treatment of Rapa could further alleviate cytotoxicity induced by the LPS related inflammation (###: *p* < .01, Rapa‐pre + LN 1 h vs. LN 1 h; †: *p* < .01, Rapa‐pre + LN 3 h vs. LN 3 h; ††: *p* < .01, Rapa‐pre + LN 6 h vs. LN 6 h). Oppositely, pre‐treatment of 3‐MA could also aggravate cytotoxicity induced by the LPS related inflammation (†††: *p* < .01, 3‐MA‐pre + LN 1 h vs. LN 1 h; *#: *p* < .01, 3‐MA‐pre + LN 3 h vs. LN 3 h; *†: *p* < .01, 3‐MA‐pre + LN 6 h vs. LN 6 h); (D) in WT cells, pre‐treatment of Rapa (1 μmol/L) for 6 h did not influence JC‐1 stained MMP (*: *p* > .05, Rapa‐pre vs. NC). Likewise, pre‐treatment of 3‐MA (50 μM) for 6 h did not influence MMP (**: *p* > .05, 3‐MA‐pre vs. NC). Comparing with NC cells, Nigericin (5 μM) for 1, 3, and 6 h after LPS (1 mg/L) resulted in time‐dependent MMP dissipation (***: *p* < .01; #: *p* < .01; ##: *p* < .01). Pre‐treatment of Rapa could significantly alleviate MMP dissipation induced by the LPS related inflammation (###: *p* < .01, Rapa‐pre + LN 1 h vs. LN 1 h; †: *p* < .01, Rapa‐pre + LN 3 h vs. LN 3 h; ††: *p* < .01, Rapa‐pre + LN 6 h vs. LN 6 h). Oppositely, pre‐treatment of 3‐MA could significantly aggravate MMP dissipation induced by the LPS related inflammation (†††: *p* < .01, 3‐MA‐pre + LN 1 h vs. LN 1 h; *#: *p* < .01, 3‐MA‐pre + LN 3 h vs. LN 3 h; *†: *p* < .01, 3‐MA‐pre + LN 6 h vs. LN 6 h); (E) in Tg‐GSDMD cells, pre‐treatment of Rapa or 3‐MA did not influence MMP (*: *p* > .05, Rapa‐pre vs. NC; **: *p* > .05, 3‐MA‐pre vs. NC). Comparing with NC cells, the trend of LN treatment induced MMP dissipation for 1, 3 h, and 6 h was recorded (***: *p* < .01; #: *p* < .01; ##: *p* < .01). Pre‐treatment of Rapa could further alleviate MMP dissipation induced by the LPS related inflammation (###: *p* < .01, Rapa‐pre + LN 1 h vs. LN 1 h; †: *p* < .01, Rapa‐pre + LN 3 h vs. LN 3 h; ††: *p* < .01, Rapa‐pre + LN 6 h vs. LN 6 h). Oppositely, pre‐treatment of 3‐MA could also aggravate MMP dissipation induced by the LPS related inflammation (†††: *p* < .01, 3‐MA‐pre + LN 1 h vs. LN 1 h; *#: *p* < .01, 3‐MA‐pre + LN 3 h vs. LN 3 h; *†: *p* < .01, 3‐MA‐pre + LN 6 h vs. LN 6 h); (F) in GSDMD‐KO cells, pre‐treatment of Rapa or 3‐MA did not influence MMP (*: *p* > .05, Rapa‐pre vs. NC; **: *p* > .05, 3‐MA‐pre vs. NC). Comparing with NC cells, the trend of LN treatment induced MMP dissipation for 1, 3, and 6 h was observed (***: *p* < .01; #: *p* < .01; ##: *p* < .01). Pre‐treatment of Rapa could further alleviate MMP dissipation induced by the LPS related inflammation (###: *p* < .01, Rapa‐pre + LN 1 h vs. LN 1 h; †: *p* < .01, Rapa‐pre + LN 3 h vs. LN 3 h; ††: *p* < .01, Rapa‐pre + LN 6 h vs. LN 6 h). Oppositely, pre‐treatment of 3‐MA could also aggravate MMP dissipation induced by the LPS related inflammation (†††: *p* < .01, 3‐MA‐pre + LN 1 h vs. LN 1 h; *#: *p* < .01, 3‐MA‐pre + LN 3 h vs. LN 3 h; *†: *p* < .01, 3‐MA‐pre + LN 6 h vs. LN 6 h). NC: negative control; Rapa: rapamycin; 3‐MA: 3‐methyladenine; LN: LPS 3 h + Nigericin; LDH: lactate dehydrogenase

Moreover, in order to further explore the cross‐talk between GSDMD induced mitochondria injury and mitophagy mediated compensatory mechanism in response to LPS related inflammation in cardiomyocytes, the pharmacological intervention on autophagy was applied to HL‐1 cardiomyocytes with or without GSDMD overexpression or knocking out. HL‐1 cells were treated by rapamycin (a widely used autophagy‐inducing agent) or 3‐methyladenine (3‐MA, a widely used autophagy‐inhibiting agent) to testify the effect of enhancement or impairment of mitophagy on cytotoxicity. Pre‐treatment (6 h before LPS plus Nigericin sequential treatment) of rapamycin or 3‐MA was given to HL‐1 cells with different genotype (WT, GSDMD‐KO, or Tg GSDMD). Lactate dehydrogenase (LDH) assay or IC‐1 staining assay was applied to measure the cytotoxicity and MMP of HL‐1 cells treated by LPS 3 h plus Nigericin (1, 3, or 6 h) with or without pre‐treatment of rapamycin or 3‐MA for 6 h (Figure 8A–C). Cytotoxicity was evaluated by measuring LDH release level in supernatant of cultured cells. Inflammatory stress increased cytotoxicity in WT HL‐1 cells. However, pre‐treatment of rapamycin could decrease cytotoxicity at early phase (Figure 8A). However, the benefit brought by pre‐treated rapamycin could be neutralized in GSDMD‐Tg cells. In GSDMD‐Tg cardiomyocytes, more severe cytotoxicity was detected, and the protective effect by autophagy agonist was weakened (Figure 8B). The cytotoxicity in GSDMD‐KO cells was significantly decreased, no matter the autophagy was strengthened or compromised (Figure 8C). Similarly, inflammation induced MMP dissipation could be aggravated by 3‐MA and inhibited by rapamycin in WT cells (Figure 8D). In Tg‐GSDMD cells, 3‐MA could further enhance the effect of inflammation mediated MMP regression. However, the protective effects of rapamycin dampened (Figure 8E). Oppositely, MMP was significantly restored with treatment of LPS 3 h plus Nigericin 6 h in GSDMD‐KO cells. Pre‐treatment of rapamycin or 3‐MA failed to further affect MMP in GSDMD‐KO cells with treatment of LPS 3 h plus Nigericin 6 h (Figure 8F). The consistency of these data collectively suggested that induced autophagy could provide protective effect in inflammatory injury.

## 3 DISCUSSION

In the present study, dual‐fluorescence labelled GSDMD (BFP‐M_GSDMD‐EYFP) as a novel tool was successfully applied to manifest the cleavage of GSDMD and spatiotemporally indicate the subcellular localization of GSDMD‐N. Tg‐GSDMD HL‐1 cardiomyocyte and GSDMD‐KO HL‐1 cardiomyocyte were constructed for further investigation. To our surprise, a time‐dependent pattern on the subcellular localization of GSDMD‐N was recorded, indicating GSDMD‐N anchoring to mitochondria to rapidly respond to the inflammatory stimulation in cardiomyocytes. Herein, dynamic changes of GSDMD‐N sub‐cellular localization were disclosed. Additionally, the pore‐forming function of GSDMD‐N was proved to be alternative regulation of MMP repression in addition to the canonical mPTP mechanism, contributing to mitochondrial dysfunction in LPS related inflammatory injury of cardiomyocyte. Mitophagy played an important role of mitochondria QC, and this explained why mitochondrial morphology and function changed little in the early phase of inflammatory stress. However, benefits originating from mitophagy mediated mitochondria QC gradually disappeared with prolonged stimulation of inflammatory stress, partly due to the repressed autophagic flux. To date, the crosstalk between mitophagy and GSDMD‐N induced mitochondrial injury during inflammatory stress was scarcely reported before. In general, our study highlights a time‐dependent pattern of the subcellular localization of GSDMD‐N, as well as crosstalk between mitophagy mediated mitochondrial QC and GSDMD‐N induced mitochondrial injury during inflammatory stress induced cardiomyocyte injury. Our study suggested that mitophagy could target to compensate GSDMD mediated mitochondrial injury. This study based on cardiomyocyte investigation has significance of clinical translational meaning to accentuate the importance of interaction between mitochondrial GSDMD and mitophagy in myocardial inflammatory disease, which has potential to be treated as pharmaceutical intervention target in inflammatory heart disease.

Mitochondrial injury underlying the pore‐forming function of GSDMD‐N and its relationship with mitophagy in inflammatory injury of HL‐1 cardiomyocyte is investigated in this study. Rogers et al. reported that GSDMD‐N generated by NLRP3‐caspase pathway could permeabilise the mitochondria to initiate the mitochondrial apoptotic pathway.^15^ In addition, another study dynamically depicted serial scenes of GSDMD‐mediated subcellular events with chronological sequence during inflammasome mediated cell pyroptosis at the single‐cell levels.^18^ Their conclusion was perfectly in harmony with what we have reported in the present study, revealing that mitochondria was preferably chosen by GSDMD‐N to target at the initial stage of inflammatory stimulation. Most recently, Huang et al. published their excellent work to disclose an important role of pore‐forming protein GSDMD in LPS activated NLRP3 inflammasome pathway, and they validated mitochondrial DNA leakage from the GSDMD formed mitochondrial pores to trigger inflammatory injury.^16^ The mitochondrial membrane acted as platform for the assembly and activation of NLRP3 inflammasome.^23^ In previous studies, mitochondria originated stimuli such as reactive oxygen species,^24^ potassium^25^ and ATP concentration^26^, ^27^ initiate GSDMD cleavage after activating NLRP3 inflammasome. However, our study in accordance with other evidences^15^, ^16^, ^18^ indicated that GSDMD‐N could oligomerize in mitochondria and further aggravate mitochondrial injury, suggesting a presumed vicious circle between GSDMD activation and mitochondria damage. Mitochondrial ultrastructure study disclosed the existence of 10‐20 nm pores in mitochondria in LPS plus Nigericin treated HL‐1 cells, and the calibre of these pores was consistent to the description of GSDMD related pore reported by Ding et al.^5^ Recent works by Xia et al. clarified the structure of the GSDMD pore as one with the outer diameter around 30 nm.^11^ The characteristics of mitochondrial pores in our study seemed to conform to findings by Ding et al. and Xia et al. In addition, we found the aforementioned pore was scarcely captured in GSDMD‐KO cells after LPS treatment, which further validate the relevance between mitochondrial pore and GSDMD. However, the pore‐like structure in mitochondria captured by TEM was not extensively distributed. It remained unclear whether this structure appeared to be real pore, or it could be other rare structure.

In this study, ablation of GSDMD failed to completely reverse the damage of mitochondria and fully recover the impaired autophagic flux, indicating other mechanism underlying in LPS induced mitochondrial injury. For instance, Haileselassie et al. reported significant mitochondrial injury which was correlating with Drp1 induced mitochondria fission in LPS‐treated H9C2 cardiomyocytes.^28^ In addition, beclin‐1 dependent autophagy also played important role in LPS‐induced septic cardiomyopathy.^29^ Therefore, inflammation induced mitochondrial stress should be regulated by multifactorial mechanism. Furthermore, we noticed that the fluorescence of GSDMD‐C fragments was concentrated and accumulated after the activation of GSDMD. Herein, we assumed that the aggregation of GSDMD‐C might facilitate the elimination of itself as a kind of non‐functional protein fragment.

Our findings suggested that mitochondria localization or cellular membrane localization of GSDMD‐N determines the fate of the cell. Mitochondria might act as sponges to buffer the over‐activated GSDMD during LPS related inflammation. After GSDMD‐N combining to mitochondria, impaired mitochondrial integrity could initiate mitochondria‐targeted autophagy. Therefore, activated mitophagy could recognize and eliminate injured parts of mitochondria. However, effects of inflammation amplification could promptly damage the balance between GSDMD‐N induced damage and mitophagy mediated QC. Accordingly, early intervention to inhibit the persistent localization of GSDMD‐N to mitochondrial membrane and enhancing the mitophagy mediated mitochondrial QC could improve the function of mitochondria and facilitate cells to survive from the inflammatory stress. Interestingly, from the latest study of Karmakar et al., the LC3 ligated autophagosome could mediate the trafficking of GSDMD‐N between organelles in neutrophil,^12^ indicating autophagosome as a carrier to facilitate the translocation of GSDMD‐N. Additionally, our study verified the fluorescent co‐localization of GSDMD‐N and LC3, and the interaction between GSDMD‐N and LC3 was validated by CO‐IP. In summary, the LC3 ligated autophagosome recognized GSDMD‐N combined mitochondrial debris to eliminate the injured mitochondria. Accordingly, mitochondria might act to buffer activated GSDMD, while mitophagy mediated mitochondrial QC could compensate GSDMD‐N induced mitochondrial injury. In this study, the expression of P62 is significantly elevated at 6 h in WT HL‐1 cells suggesting restrained autophagic flux. In contrast, P62 is decreased at 6 h in GSDMD‐KO HL‐1 cells, indicating restored autophagic flux. This seemed to be contradictory with the notion that mitochondrial injury by GSDMD‐N leading to mitophagy. We assumed that GSDMD mediated mitochondria injury could directly induce mitophagy by interaction of GSDMD with LC3B. Enhanced mitophagy act as compensatory mechanism to protect cells from inflammatory injury. However, persistently existed injury factors could weaken the mitophagy mediated protective effects by weakening the autophagic flux, and that is why P62 level is increased again at 6 h in WT cells. Oppositely, the restored autophagic flux was observed in GSDMD‐KO HL‐1 cells in early phase of LPS related inflammation, and this might be attributed to other compensatory mechanism instead of the interaction between GSDMD and LC3B. It is thought‐provoking to panoramically exhibit the intra‐cellular transport of GSDMD‐N. GSDMD‐N was hypothesized to potentially target other organelles, such as Golgi body, lysosome and endoplasmic reticulum (ER), subsequently influencing their function. To validate this hypothesis, the dual‐fluorescence labelled GSDMD in alliance with other organelle specific staining, such as Lyso‐Tracker for lysosome, ER‐Tracker for endoplasmic reticulum^30^ and Golgi‐Tracker for Golgi body,^31^ could be used to indicate the co‐localization of GSDMD‐N and a certain organelle. It was reported that the recruitment of NLRP3 to Golgi network could lead to NLRP3 aggregation and activation in response to inflammatory stimuli^32^ and the NLRP3 related GSDMD translocation through Golgi network might be further disclosed by the fluorescence tracking involved in the present study.

In all, results attained from this study correlate with heart disease such as sepsis‐induced cardiomyopathy, infectious endocarditis, myocarditis, or some systemic inflammatory disease which could lead to cardiac involvement. We are looking forward to testifying the results originating from this study in some more specific heart disease models in future to develop the potential target for pharmaceutical intervention.

### 3.1 Limitations

Fluorescence of nuclei was failed to be labelled in BFP‐M_GSDMD‐EYFP expressed cells, attributed to much overlap of the excitation and emission spectrum between nuclear staining dyes (DAPI or Hoechst) and BFP. However, the bright‐field picture was acquired simultaneously to show the cellular contour and nuclei. If investigation of co‐localization with nuclei is involved in future, other combination of fluorescent protein labels such as GFP and mCherry double‐labelled GSDMD could be applied to solve this problem. CsA as a well‐recognized mPTP inhibitor was used to depress the function of mPTP in this study, however, gene knocking‐out or RNA interference technique based mPTP ablation could facilitate to acquire more reliable conclusion than pathological intervention. The pore‐like structure in mitochondria captured by TEM was scarcely observed in its quantity. It remained unclear whether this structure appeared to be real pore, or it could be other rare structure. Additional investigation should be more focused to clarify its essence.

## 4 CONCLUSION

In the present study, dual‐fluorescence labelled GSDMD (BFP‐M_GSDMD‐EYFP) as a novel tool to manifest the cleavage of GSDMD and spatiotemporally could indicate the subcellular localization of GSDMD‐N. Herein, dynamic changes of GSDMD‐N in mitochondria and cytoplasmic localization were disclosed. A time‐dependent pattern on the subcellular localization of GSDMD‐N suggested mitochondria anchoring of GSDMD‐N as a rapid response to inflammatory stress. Moreover, the pore‐forming function of GSDMD‐N could be a complementary mechanism of MMP repression, in addition to the canonical mPTP pore. Our study suggested that mitochondrial autophagy specifically targeted to compensate GSDMD mediated mitochondrial injury. This study based on cardiomyocyte investigation has significance of clinical translational meaning to accentuate the importance of interaction between mitochondrial GSDMD and mitophagy in myocardial inflammatory disease. Our findings support pharmaceutical intervention on enhancing autophagy or inhibiting GSDMD as potential target for inflammatory heart disease treatment.

## 5 MATERIALS AND METHODS

### 5.1 Cell and intervention

The mouse myocardial originated HL‐1 cells and human originated HEK‐293T cells were purchased from the cell bank of Chinese Academy of Science. Cells were cultured in Dulbecco's Modified Eagle Medium (DMEM) medium which was enriched with 10% fetal bovine serum (FBS, Gibco, Australia). Inflammatory stress was induced by LPS and Nigericin (Nig) sequential treatment. LPS (Sigma‐Aldrich, L2880) with concentration of 1 mg/L was used for priming of inflammation, and Nig (Merck, 481990) with concentration of 5 μM was used for inflammation activation. CCCP with concentration of 50 mM was used for positive control of dissipated mitochondria membrane potential in JC‐1 staining experiment. Cyclosporine A (CsA) as small molecule inhibitor purchased from MedChemExpress with concentration of 50 nM was used to inhibit the mPTP. MCC950 (MedChemExpress) as the inhibitor of NLRP3 inflammasome was used for inhibiting inflammatory stress (with concentration of 10 nM). Pre‐treatment of rapamycin (Rapa) purchased from MedChemExpress was used to induce autophagy (1 μmol/L), and pre‐treatment of 3‐Methyladenine (3‐MA) was used to inhibit autophagy (50 μM). Cytotoxicity is detected by LDH release using LDH Cytotoxicity Assay Kit (C0017, Beyotime Biotechnology, China) to measure the OD value of the absorbance in supernatant of cultured cells at 490 nm.

### 5.2 Construction of recombinant BFP‐M_GSDMD‐EYFP overexpression plasmid vector

The nucleotide sequence of EYFP and BFP were ligated to the C‐terminal and N‐terminal of mouse originated GSDMD gene sequence, respectively, to construct the fusion gene of BFP‐M_GSDMD‐EYFP. CMV‐MCS‐PGK‐Puro lentiviral over‐expression plasmid system with length of 8065 base pair (bp) was used as the vector of the target gene (Figure 1A). The lentiviral plasmid was processed by restriction endonuclease, and the sequence of BFP‐M_GSDMD‐EYFP was inserted into the plasmid vector (Figure 1B). Sequencing results of the re‐constructed overexpression plasmid vector validated that the sequence of the inserted gene in the recombinant clone was completely consistent with that of the target gene, indicating the successfully constructed plasmid. The constructed overexpression vector was transfected into HEK‐293T cells by HG transgene reagent (Genomeditech, TG‐10012‐S), and the overexpression of BFP‐M_GSDMD‐EYFP fusion protein was verified by WB by anti‐GSDMD antibody (ab209845) at ∼110 kD (BFP: 29 kD; EYFP: 29 kD; and GSDMD: 53 kD). Recombinant overexpression plasmid vector was then packaged into lentivirus, and HEK‐293T cells were transfected by packaged lentivirus. The fluoroscopic microscope (Leica DMLB2) was used to detect the fluorescence intensity of the infected HEK‐293T cells with different rate of dilution to determine the virus titre (the number of fluorescence positive cells multiplied by the corresponding dilution rate). HL‐1 cells were infected by PGMLV‐CMV‐BFP‐M_GSDMD‐EYFP‐PGK‐Puro lentivirus and cultured for five days. Then processed eukaryotic resistance screening, based on the puro gene in the plasmid providing drug‐resistance to the antibiotics puromycin (total lethal concentration: 2 μg/ml and growth maintenance concentration: 1 μg/ml), could purify the BFP‐M_GSDMD‐EYFP stably expressed HL‐1 cell. BFP‐M_GSDMD‐EYFP transgenic HL‐1 cell line was ultimately verified by WB. The BFP‐M_GSDMD‐EYFP fusion protein was detected at ∼110 kD, while the innate GSDMD was detected at 53 kD (Figure 1E).

### 5.3 Construction of CRISPR/CAS9 based GSDMD knocking‐out cell line

The packaged lentiCas9‐Blast lentivirus was used to infect the HL‐1 cell. After culturing for 5 days, the processed eukaryotic resistance screening, based on the blast gene in the plasmid providing drug‐resistance to the antibiotics blasticidin (total lethal concentration: 6 μg/ml and growth maintenance concentration: 3 μg/ml), could purify the BFP‐M_GSDMD‐EYFP stably expressed HL‐1 cell. Then, the mixture of 1% penicillin/streptomycin plus 3 μg/ml blasticidin was treated for the growth of maintenance to acquire the Cas9‐Blast HL‐1 cell line. The sequence of CRISPR guide RNA (gRNA) was designed and synthesized based on the sequence of mouse GSDMD. Three candidate gRNA targeted sequence in GSDMD (AATGTGATCAAGGAGGTAAG; CCTGTCAATCAAGGACATCC; CCTGGAGCCCAGTGCTCCAG) and one control RNA sequence in GSDMD (ACGGAGGCTAAGCGTCGCAA) were selected. Corresponding three pairs of gRNA oligomeric single stranded DNA sequence were designed and synthesized as primers. The oligomeric single stranded DNA was annealed to form double strands, and the double stranded gRNA oligo was indirectly inserted into the packaged Cas9‐Blast vector by restriction endonuclease (BsmBI) to construct CRISPR/CAS9 recombinant plasmid. Then the inserted sequence in the recombinant clone was compared with the designed oligo sequence by sequencing comparison to validate the successfully constructed lentivirus vector. Afterwards, HEK‐293T cells were transfected with the constructed lentivirus vector with different gRNA of five dilution rates (10^−1^, 10^−2^, 10^−3^, 10^−4^ and 10^−5^). The real‐time PCR was used to measure the tire of lentivirus to make sure the titre of the lentivirus >1 × 10^10^ TU/ml. The stable strain of Cas9‐Blast HL‐1 cells was infected with three different packed GSDMD‐sgRNA lentivirus sequence to obtain three lines of Cas9‐GSDMD sgRNA HL‐1 cells. Then the DNA in each cell line was extracted for PCR amplification (primer‐F: CTTCCTCCTAAGTGCTGTGGC; primer‐R: GATTGCTCACAACCATCTGTCC), and the sequence of amplified product was investigated to find out the optimal result. Moreover, the level of GSDMD ablation at protein level was also testified by WB.

### 5.4 Establishment of the BFP‐M_GSDMD‐EYFP fusion proteins stably expressed cell line and CRISPR‐CAS9 based GSDMD‐KO cell line

The information of the lentivirus vector was shown in Figure SA. The schematic diagram of the BFP‐M_GSDMD‐EYFP plasmid was illustrated in Figure SB. To validate the protein‐expressing function of the BFP‐M_GSDMD‐EYFP plasmid, HEK‐293T cells were transfected with this plasmid and lentivirus vector by HG transgene reagent (TG‐10012‐S), and the fusion protein of BFP‐M_GSDMD‐EYFP (BFP: 29 kD; EYFP: 29 kD; and GSDMD: 53 kD) was detected by anti‐GSDMD antibody at ∼110 kD (Figure SC). Because the primary anti‐GSDMD antibody was originated from mouse, the endogenous GSDMD expression of HEK‐293T cells (human origin) at ∼60 kD cannot be detected. Besides, the fusion fluorescence of EYFP and BFP can be observed after transfection in different viral titre (Figure SD). The lentivirus was packaged based on previously mentioned method. Then the HL‐1 cell was infected with packaged virus. Compared to the concentration of lentivirus solution with 10 × 10^−7^/ml, the concentration of lentivirus solution with 10 × 10^−3^/ml could attain ideal transfect efficiency. The optimal titre of lentivirus was 5 × 10^9^ TU/ml. The BFP‐M_GSDMD‐EYFP stably expressed strain was isolated by repeated resistance screening. In HL‐1 cells, the fusion protein was also detected at ∼110 kD except for the innate GSDMD expression at 53 kD (Figure SE), by a mouse originated primary antibody (ab209845). Oppositely, neither HEK‐293T cells nor HL‐1 cells showed fusion protein expression in scrambled sequence packed lentivirus group (Figures SC and SE). The information of the lentivirus vector for sgRNA was shown in Figure SF. Three different sgRNA (sgRNA1, sgRNA2 and sgRNA3) were designed, and sgRNA2 mediated GSDMD knocking out was validated by western blotting (Figure SG). Seven independent single cells were isolated and cultured to develop into different cell strains, in which GSDMD‐KO at protein level was verified in five cell strains (Figure SH). Afterwards, DNA was extracted from each cell strain for sequence alignment with the original sequence in WT strain. At last, one cell strain was chosen as stable cell line of GSDMD‐KO for subsequent experiments.

### 5.5 Laser confocal microscopy

Cultured cells with fluorescence protein label or stained by exogenous fluorescence dye could be recorded by Laser confocal microscopy (Olympus, FLUOVIEW FV3000) with high resolution to assess the intracellular GSDMD translocation, autophagosome, mitophagosome and autophagic flux. The BFP‐GSDMD‐EYFP fusion protein was detected by two channels of wave length (Alexa fluor 488 and Alexa fluor 532). GSDMD‐N was identified by Alexa fluor 532 channel, and GSDMD‐C was recognized by Alexa fluor 488 channel. Mitochondria was visualized through MitoTracker Deep Red (Invitrogen, M22426) dye staining by Alexa fluor 647 channel. The nuclei were detected after Hoechst dye staining by Hoechst33258 channel. The double‐labelled mCherry‐GFP LC3B (Beyotime C3011) was detected by Alexa fluor 568 channel and mCherry channel to compare the changes of the ratio of yellow fluorescence and red fluorescence. Therefore, the autophagic process can be tracked very effectively by fusion expression of mCherry‐GFP‐LC3B protein. If mCherry‐GFP‐LC3B existed in the cytoplasm in the form of diffuse yellow fluorescence (combined effect of mCherry and GFP), prohibited autophagic flux was indicated. After the fusion of autophagosome and lysosome, red dots increased due to the partial quenching of GFP fluorescence indicating unimpeded autophagic flux.

### 5.6 Transmission electron microscopy

To prepare the TEM sample, the cell pellet was collected and fixed in 2.5% glutaraldehyde overnight. Then, the sample was dehydrated by gradient ethanol, embedded with pure acetone, solidified in oven, sliced and stained with gold. The electron‐microscopic system (FEI Tecnai G2 Spirit) was used to acquire TEM images of cell. Mitochondria morphology, autophagosome, mitophagosome and autolysosome were assessed by TEM.

### 5.7 Western blots

Different cell fraction including total cell lysate, mitochondria lysate or cytosol fraction were prepared, respectively. The radio immunoprecipitation assay (RIPA) lysis buffer were added to each cell fraction with mixture of protease inhibitor cocktail. The concentration of proteins in different sample was measured by BCA (bicinchoninic acid) assay kit method. Total protein was separated on a 12% SDS‐PAGE, and then the gel was transferred to polyvinylidene difluoride membranes. Afterwards, the membrane was diluted by TBS and blocked by skim milk. Then, membranes were incubated with primary antibodies of GSDMD (1:1000, ab209845, Abcam), cleaved Caspase‐1 p20 (1:1000, 4199, CST), LC3B (1:1000, 2775, CST), sequestosome‐1 (SQSTM1/p62) (1:1000, 5114, CST), COX‐4 (1:1000, CST), cleaved GSDMD‐N (1:1000, Abcam, EPR20829‐408), β‐Actin (1:5000, 12620, CST), GAPDH (1:5000, 8884, CST), Parkin (1:1000, 4211, CST), or Pink1 (1:1000, ab23707, abcam) at 4°C overnight. Non‐horseradish peroxidase (HRP) conjugated primary antibody was incubated with HRP‐conjugated secondary antibody and the protein‐antibody binding reaction in membrane was detected by the enhanced chemiluminescence assays.

### 5.8 Co‐immunoprecipitation

Thermo Pierce Crosslink IP Kit (Thermo Scientific, 26149) was used for CO‐IP experiment. GSDMD and its cleaved fragment GSDMD‐N, together with LC3B, were captured from cell lysate using GSDMD antibody (ab209845) or LC3B antibody (Abcam, EPR18709). Mouse IgG antibodies for IP (Biotin) (ab131367) and rabbit IgG antibodies for IP (Cell Signalling Technology, 3678S) were used as control for excluding non‐specific binding reaction. HL‐1 cells with or without LPS plus Nigericin treatment were harvested, homogenized and further re‐suspended in lysis buffer. After incubating on ice and high‐speed centrifuging, the supernatant was quantified by BCA protein assay kit (P0012). The cell lysate was immediately precleared by Protein A/G PLUS‐Agarose (Santa Cruz Biotechnology, sc‐2003) at 4°C for 1 h. The precleared lysate was then co‐immunoprecipitated overnight at 4 °C with anti‐GSDMD or anti‐LC3B antibody covalently coupled to Protein A/G PLUS‐Agarose. For the control experiment, the same amount of lysate was incubated with mouse IgG or rabbit IgG covalently coupled with Agarose. After the last centrifuge step, protein complexes were washed out from the beads at 95 °C for 5 min.

### 5.9 Mitochondria fraction isolation

The protocol of mitochondria isolation and MMP measurement was as follows: (i) Mitochondria were isolated from HL‐1 cells following instructions of the tissue mitochondria isolation kit (C3606, Beyotime, China). Cultured cells were digested by trypsin (Gibco), and the homogenate was centrifuged at 1000× *g* for 5 min at 4°C. The supernatant was carefully removed, and the homogenate was centrifuged again at 3500× *g* for 10 min at 4°C. The pellet was purified mitochondria, and the cytosolic protein was collected from the supernatant after another centrifugation at 12000×g for 10 min.

### 5.10 Mitochondrial membrane potential measurement

MMP was measured by JC‐1 staining assay kit. JC‐1 polymer (excitation: 525 nm; emission: 590 nm) indicating the polarized MMP could emit red fluorescence. JC‐1 monomer (excitation: 490 nm; emission: 530 nm) indicating the depolarized MMP could emit green fluorescence. Changes of MMP were assessed by the ratio of fluorescence intensity between green colour and red colour. MMP was measured in cultured cell by JC‐1 assay kit (C2006, Beyotime, China). Cultured cells were incubated with JC‐1 stains for 20 min. Afterwards, fluorescence microplate reader (FlexStation3, USA) was used to record the intensity of fluorescence of JC‐1 stains. Fluorescence image was captured by fluorescence microscope system (Leica DMLB2).

### 5.11 Statistics

Mean ± standard error (SE) was used as estimate for continuous variable. Difference between two groups was assessed by student's *t* test. In addition, for comparison among more than three groups, inter‐group difference was compared by one‐way analysis of variance (ANOVA). Statistical analysis was made via SPSS software 19.0. A value of *p* < .05 was considered to be significant.

## CONFLICT OF INTEREST

The authors declare no conflict of interest.

## Supporting information

Supporting Information

Supporting Information
