# Supplementary material for: Translocation of gasdermin D induced mitochondrial injury and mitophagy mediated quality control in lipopolysaccharide related cardiomyocyte injury
Source: Clin Transl Med. 2022 Aug 28;12(8):e1002. doi: 10.1002/ctm2.1002 (PMC9420421; doi:10.1002/ctm2.1002)
Supplement: Supplementary file 1 — Supporting Information [file CTM2-12-e1002-s002.doc]

**Supplemental material**

**Establishment of the BFP-M_GSDMD-EYFP fusion proteins stably expressed cell line and CRISPR-CAS9 based GSDMD KO cell line**

The information of the lentivirus vector was shown in Supplemental figure A. The schematic diagram of the BFP-M_GSDMD-EYFP plasmid was illustrated in Supplemental figure B. To validate the protein-expressing function of the BFP-M_GSDMD-EYFP plasmid, HEK-293T cells were transfected with this plasmid and lentivirus vector by HG transgene reagent (TG-10012-S) reagent, and the fusion protein of BFP-M_GSDMD-EYFP (BFP: 29 kD; EYFP: 29 kD; and GSDMD: 53 kD) was detected by anti-GSDMD antibody at ~110kD (Supplemental figure C). Because the primary anti-GSDMD antibody was originated from mouse, the endogenous GSDMD expression of HEK-293T cells (human origin) at ~60kD cannot be detected. Besides, the fusion fluorescence of EYFP and BFP can be observed after transfection in different viral titre (Supplemental figure D). The lentivirus was packaged based on previously mentioned method. Then the HL-1 cell was infected with packaged virus. Compared to the concentration of lentivirus solution with 10*10-7/ml, the concentration of lentivirus solution with 10*10-3/ml could attain ideal transfect efficiency. And the optimal titre of lentivirus was 5*107 TU/ml. The BFP-M_GSDMD-EYFP stably expressed strain was isolated by repeated resistance screening. In HL-1 cells, the fusion protein was also detected at ~110 kD except for the innate GSDMD expression at 53 kD (Supplemental figure E), by a mouse originated primary antibody (ab209845). Oppositely, neither HEK-293T cells nor HL-1 cells did show fusion protein expression in scrambled sequence packed lentivirus group (Supplemental figure C and E). The information of the lentivirus vector for sgRNA was shown in Supplemental figure F. 3 different sgRNA (sgRNA1, sgRNA2, and sgRNA3) were designed, and sgRNA2 mediated GSDMD knocking out was validated by western blotting (Supplemental figure G). 7 independent single cells were isolated and cultured to develop into different cell strains, in which GSDMD KO at protein level was verified in 5 cell strains (Supplemental figure H). Afterwards, DNA was extracted from each cell strains for sequence alignment with the original sequence in WT strain. At last, 1 cell strain was chosen as stable cell line of GSDMD KO for subsequent experiments.
