# Supplementary figures and images for: Translocation of gasdermin D induced mitochondrial injury and mitophagy mediated quality control in lipopolysaccharide related cardiomyocyte injury
Source: Clin Transl Med. 2022 Aug 28;12(8):e1002. doi: 10.1002/ctm2.1002 (PMC9420421; doi:10.1002/ctm2.1002)

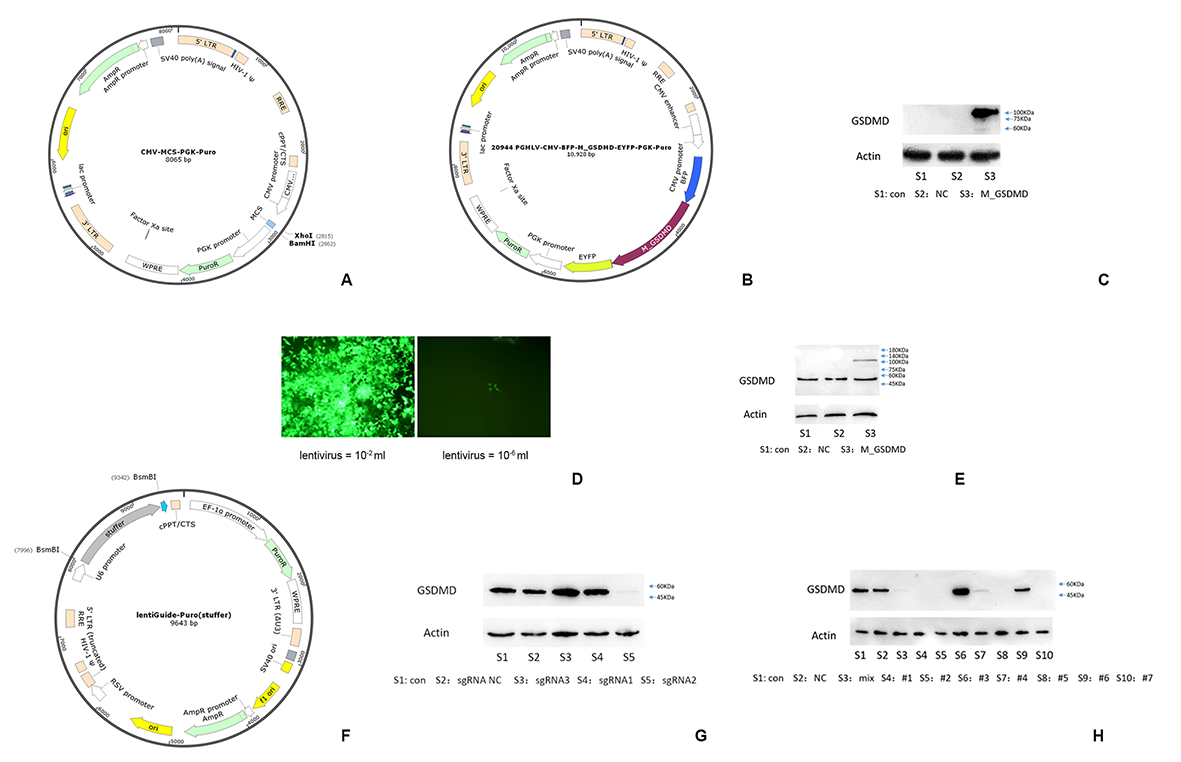

Supplement: Supplementary file 2 — Supporting Information [file CTM2-12-e1002-s001.tif]
